# Structural insights into the *Escherichia coli* lysine decarboxylases and molecular determinants of interaction with the AAA+ ATPase RavA

**DOI:** 10.1038/srep24601

**Published:** 2016-04-15

**Authors:** Eaazhisai Kandiah, Diego Carriel, Julien Perard, Hélène Malet, Maria Bacia, Kaiyin Liu, Sze W. S. Chan, Walid A. Houry, Sandrine Ollagnier de Choudens, Sylvie Elsen, Irina Gutsche

**Affiliations:** 1 University Grenoble Alpes, IBS, F-38044 Grenoble, France; 2 CNRS, IBS, F-38044 Grenoble, France; 3 CEA, IBS, F-38044 Grenoble, France; 4 INSERM, Biologie du Cancer et de l’Infection (UMR-S1036), F-38054 Grenoble, France; 5 Centre National de la Recherche Scientifique (CNRS), ERL5261 F-38054 Grenoble, France; 6 University Grenoble Alpes, BIG, Grenoble, F-38041, France; 7 CEA, BIG, Grenoble, France; 8 CNRS, LCMB, BIG, Grenoble, France; 9 Department of Biochemistry, University of Toronto, Toronto, Ontario M5S 1A8, Canada

## Abstract

The inducible lysine decarboxylase LdcI is an important enterobacterial acid stress response enzyme whereas LdcC is its close paralogue thought to play mainly a metabolic role. A unique macromolecular cage formed by two decamers of the *Escherichia coli* LdcI and five hexamers of the AAA+ ATPase RavA was shown to counteract acid stress under starvation. Previously, we proposed a pseudoatomic model of the LdcI-RavA cage based on its cryo-electron microscopy map and crystal structures of an inactive LdcI decamer and a RavA monomer. We now present cryo-electron microscopy 3D reconstructions of the *E. coli* LdcI and LdcC, and an improved map of the LdcI bound to the LARA domain of RavA, at pH optimal for their enzymatic activity. Comparison with each other and with available structures uncovers differences between LdcI and LdcC explaining why only the acid stress response enzyme is capable of binding RavA. We identify interdomain movements associated with the pH-dependent enzyme activation and with the RavA binding. Multiple sequence alignment coupled to a phylogenetic analysis reveals that certain enterobacteria exert evolutionary pressure on the lysine decarboxylase towards the cage-like assembly with RavA, implying that this complex may have an important function under particular stress conditions.

Enterobacterial inducible decarboxylases of basic amino acids lysine, arginine and ornithine have a common evolutionary origin and belong to the α-family of pyridoxal-5′-phosphate (PLP)-dependent enzymes^1^,^2^. They counteract acid stress experienced by the bacterium in the host digestive and urinary tract, and in particular in the extremely acidic stomach^3^,^4^. Each decarboxylase is induced by an excess of the target amino acid and a specific range of extracellular pH, and works in conjunction with a cognate inner membrane antiporter. Decarboxylation of the amino acid into a polyamine is catalysed by a PLP cofactor in a multistep reaction^1^,^2^ that consumes a cytoplasmic proton and produces a CO_2_ molecule passively diffusing out of the cell, while the polyamine is excreted by the antiporter in exchange for a new amino acid substrate. Consequently, these enzymes buffer both the bacterial cytoplasm and the local extracellular environment^5^. These amino acid decarboxylases are therefore called acid stress inducible or biodegradative to distinguish them from their biosynthetic lysine and ornithine decarboxylase paralogs catalysing the same reaction but responsible for the polyamine production at neutral pH.

Inducible enterobacterial amino acid decarboxylases have been intensively studied since the early 1940^6^,^7^ because the ability of bacteria to withstand acid stress can be linked to their pathogenicity in humans. In particular, the inducible lysine decarboxylase LdcI (or CadA) attracts attention due to its broad pH range of activity and its capacity to promote survival and growth of pathogenic enterobacteria such as *Salmonella enterica* serovar Typhimurium, *Vibrio cholerae* and V*ibrio vulnificus* under acidic conditions^5^,^8^,^9^. Furthermore, both LdcI and the biosynthetic lysine decarboxylase LdcC of uropathogenic *Escherichia coli* (UPEC) appear to play an important role in increased resistance of this pathogen to nitrosative stress produced by nitric oxide and other damaging reactive nitrogen intermediates accumulating during the course of urinary tract infections (UTI)^10^,^11^. This effect is attributed to cadaverine, the diamine produced by decarboxylation of lysine by LdcI and LdcC, that was shown to enhance UPEC colonisation of the bladder^11^. In addition, the biosynthetic *E. coli* lysine decarboxylase LdcC, long thought to be constitutively expressed in low amounts, was demonstrated to be strongly upregulated by fluoroquinolones via their induction of RpoS^12^,^13^. A direct correlation between the level of cadaverine and the resistance of *E. coli* to these antibiotics commonly used as a first-line treatment of UTI could be established^12^. Both acid pH and cadaverine induce closure of outer membrane porins thereby contributing to bacterial protection from acid stress, but also from certain antibiotics, by reduction in membrane permeability^14^,^15^,^16^.

The crystal structure of the *E. coli* LdcI^17^ as well as its low resolution characterisation by electron microscopy^17^,^18^,^19^ (EM) showed that it is a decamer made of two pentameric rings. Each monomer is composed of three domains – an N-terminal wing domain (residues 1–129), a PLP-binding core domain (residues 130–563), and a C-terminal domain (CTD, residues 564–715). Monomers tightly associate via their core domains into 2-fold symmetrical dimers with two complete active sites, and further build a toroidal D5-symmetrical structure held by the wing and core domain interactions around the central pore, with the CTDs at the periphery.

Ten years ago^19^ we showed that the *E. coli* AAA+ ATPase RavA, involved in multiple stress response pathways^19^,^20^,^21^,^22^, tightly interacted with LdcI but was not capable of binding to LdcC. We described how two double pentameric rings of the LdcI^17^ tightly associate with five hexameric rings of RavA^21^ to form a unique cage-like architecture that enables the bacterium to withstand acid stress even under conditions of nutrient deprivation eliciting stringent response^19^,^21^,^23^. Furthermore, we recently solved the structure of the *E. coli* LdcI-RavA complex by cryo-electron microscopy (cryoEM) and combined it with the crystal structures of the individual proteins^23^. This allowed us to make a pseudoatomic model of the whole assembly, underpinned by a cryoEM map of the LdcI-LARA complex (with LARA standing for LdcI associating domain of RavA), and to identify conformational rearrangements and specific elements essential for complex formation^23^. The main determinants of the LdcI-RavA cage assembly appeared to be the N-terminal loop of the LARA domain of RavA and the C-terminal β-sheet of LdcI^23^.

In spite of this wealth of structural information, the fact that LdcC does not interact with RavA, although the two lysine decarboxylases are 69% identical and 84% similar^19^,^24^, and the physiological significance of the absence of this interaction remained unexplored. To solve this discrepancy, in the present work we provided a three-dimensional (3D) cryoEM reconstruction of LdcC and compared it with the available LdcI and LdcI-RavA structures. Given that the LdcI crystal structures were obtained at high pH where the enzyme is inactive (LdcI_i_, pH 8.5), whereas the cryoEM reconstructions of LdcI-RavA and LdcI-LARA were done at acidic pH optimal for the enzymatic activity, for a meaningful comparison, we also produced a 3D reconstruction of the LdcI at active pH (LdcI_a_, pH 6.2). This comparison pinpointed differences between the biodegradative and the biosynthetic lysine decarboxylases and brought to light interdomain movements associated to pH-dependent enzyme activation and RavA binding, notably at the predicted RavA binding site at the level of the C-terminal β-sheet of LdcI. Consequently, we tested the capacity of cage formation by LdcI-LdcC chimeras where we interchanged the C-terminal β-sheets in question. Finally, we performed multiple sequence alignment of 22 lysine decarboxylases from *Enterobacteriaceae* containing the *ravA-viaA* operon in their genome. Remarkably, this analysis revealed that several specific residues in the above-mentioned β-sheet, independently of the rest of the protein sequence, are sufficient to define if a particular lysine decarboxylase should be classified as an “LdcC-like” or an “LdcI-like”. Moreover, this classification perfectly agrees with the genetic environment of the lysine decarboxylase genes. This fascinating parallelism between the propensity for RavA binding and the genetic environment of an enterobacterial lysine decarboxylase, as well as the high degree of conservation of this small structural motif, emphasize the functional importance of the interaction between biodegradative enterobacterial lysine decarboxylases and the AAA+ ATPase RavA.

## Results and Discussion

### CryoEM 3D reconstructions of LdcC, LdcI_a_ and LdcI-LARA

**Figure 1:**
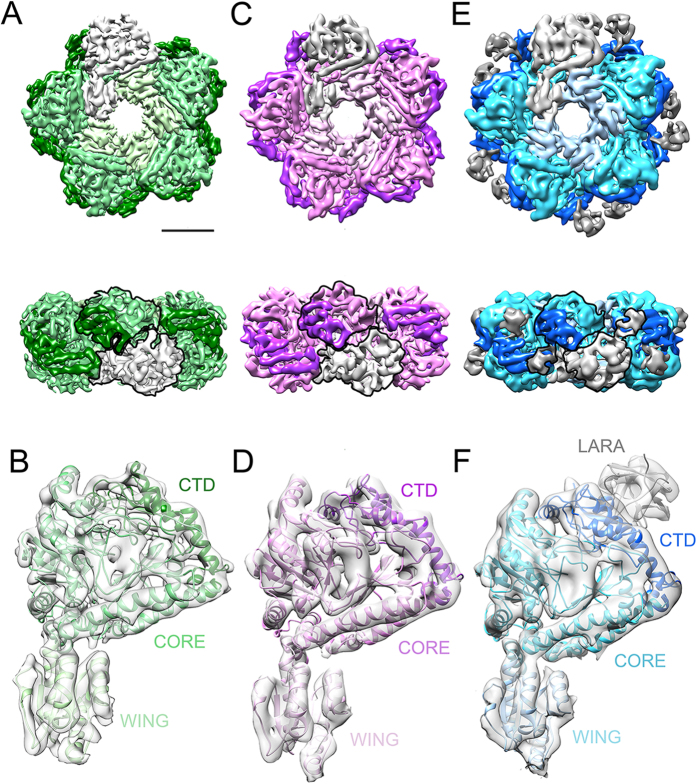
3D cryoEM reconstructions of LdcC, LdcI-LARA and LdcI_a._ (**A,C,E**) cryoEM map of the LdcC (**A**), LdcI_a_ (**C**) and LdcI-LARA (**E**) decamers with one protomer in light grey. In the rest of the protomers, the wing, core and C-terminal domains are colored from light to dark in shades of green for LdcC (**A**), pink for LdcI_a_ (**C**) and blue for LdcI in LdcI-LARA (**E**). In (**E**), the LARA domain density is shown in dark grey. Two monomers making a dimer are delineated. Scale bar 50 Å. (**B,D,F**) One protomer from the cryoEM map of the LdcC (**B**), LdcI_a_ (**D**) and LdcI-LARA (**F**) in light grey with the pseudoatomic model represented as cartoons and colored as the densities in (**A,C,E**). Each domain is indicated for clarity. Scale bar 50 Å. See also Figs S1 and S3.

In the frame of this work, we produced two novel subnanometer resolution cryoEM reconstructions of the *E. coli* lysine decarboxylases at pH optimal for their enzymatic activity – a 5.5 Å resolution cryoEM map of the LdcC (pH 7.5) for which no 3D structural information has been previously available (Figs 1A,B and S1), and a 6.1 Å resolution cryoEM map of the LdcI_a_, (pH 6.2) (Figs 1C,D and S2). In addition, we improved our earlier cryoEM map of the LdcI-LARA complex from 7.5 Å to 6.2 Å resolution (Figs 1E,F and S3). Based on these reconstructions, reliable pseudoatomic models of the three assemblies were obtained by flexible fitting of either the crystal structure of LdcI_i_ or a derived structural homology model of LdcC (Table S1). Significant differences between these pseudoatomic models can be interpreted as movements between specific biological states of the proteins as described below.

### The wing domains as a stable anchor at the center of the double-ring

**Figure 2:**
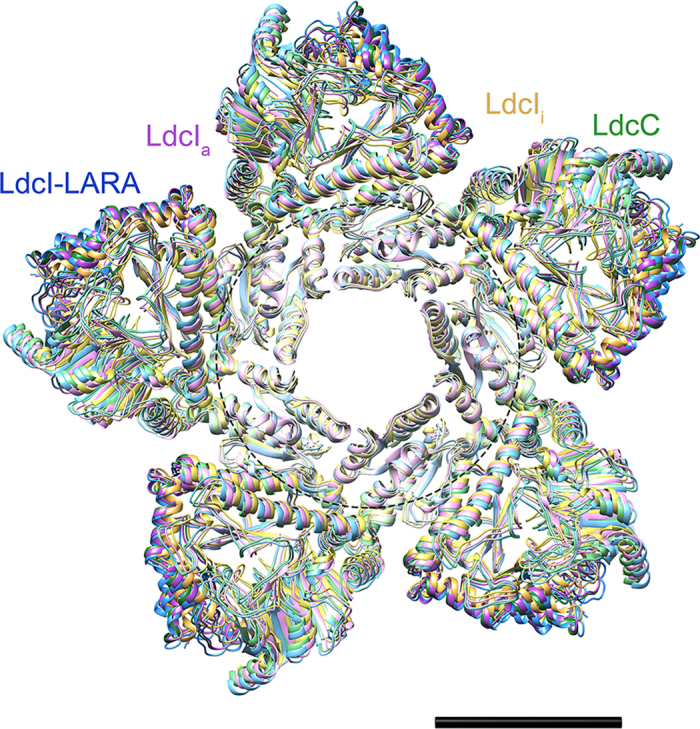
Analysis of conformational rearrangements. Superposition of the pseudoatomic models of LdcC, LdcI from LdcI-LARA and LdcI_a_ colored as in Fig. 1, and the crystal structure of LdcI_i_ in shades of yellow. Only one of the two rings of the double toroid is shown for clarity. The dashed circle indicates the central region that remains virtually unchanged between all the structures, while the periphery undergoes visible movements. Scale bar 50 Å.

As a first step of a comparative analysis, we superimposed the three cryoEM reconstructions (LdcI_a_, LdcI-LARA and LdcC) and the crystal structure of the LdcI_i_ decamer (Fig. 2 and Movie S1). This superposition reveals that the densities lining the central hole of the toroid are roughly at the same location, while the rest of the structure exhibits noticeable changes. Specifically, at the center of the double-ring the wing domains of the subunits provide the conserved basis for the assembly with the lowest root mean square deviation (RMSD) (between 1.4 and 2 Å for the Cα atoms only), whereas the peripheral CTDs containing the RavA binding interface manifest the highest RMSD (up to 4.2 Å) (Table S2). In addition, the wing domains of all structures are very similar, with the RMSD after optimal rigid body alignment (RMSD_min_) less than 1.1 Å. Thus, taking the limited resolution of the cryoEM maps into account, we consider that the wing domains of all the four structures are essentially identical and that in the present study the RMSD of less than 2 Å can serve as a baseline below which differences may be assumed as insignificant. This preservation of the central part of the double-ring assembly may help the enzymes to maintain their decameric state upon activation and incorporation into the LdcI-RavA cage.

### The core domain and the active site rearrangements upon pH-dependent enzyme activation and LARA binding

**Figure 3:**
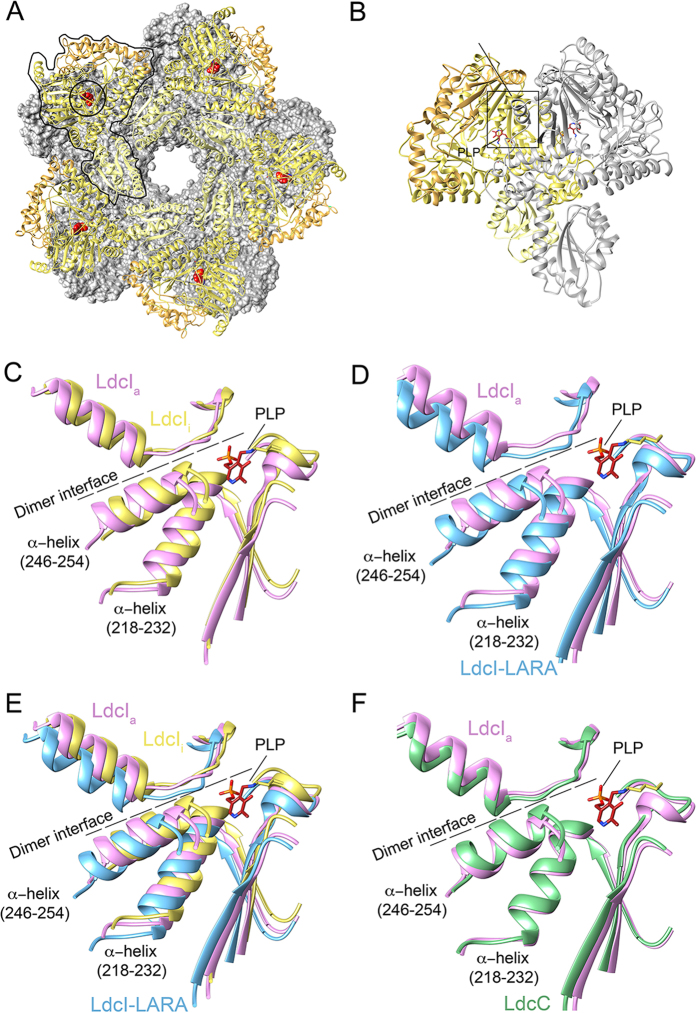
Conformational rearrangements in the enzyme active site. (**A**) LdcI_i_ crystal structure, with one ring represented as a grey surface and the second as a cartoon. A monomer with its PLP cofactor is delineated. The PLP moieties of the cartoon ring are shown in red. (**B**) The LdcI_i_ dimer extracted from the crystal structure of the decamer. One monomer is colored in shades of yellow as in Figs 1 and 2, while the monomer related by C2 symmetry is grey. The PLP is red. The active site is boxed. (**C–F**) Close-up views of the active site. The PLP moiety in red is from the LdcI_i_ crystal structure. We did not attempt to model it in the cryoEM maps. The dimer interface is shown as a dashed line and the active site α-helices mentioned in the text are highlighted. (**C**) Compares LdcI_i_ (yellow) and LdcI_a_ (pink), (**D**) compares LdcI_a_ (pink) and LdcI-LARA (blue), and (**E**) compares LdcI_i_ (yellow), LdcI_a_ (pink) and LdcI-LARA (blue) simultaneously in order to show the progressive shift described in the text. (**F**) Shows the similarity between LdcI_a_ and LdcC at the level of the secondary structure elements composing the active site. Colors are as in the other figures.

Both visual inspection (Fig. 2) and RMSD calculations (Table S2) show that globally the three structures at active pH (LdcI_a_, LdcI-LARA and LdcC) are more similar to each other than to the structure determined at high pH conditions (LdcI_i_). The decameric enzyme is built of five dimers associating into a 5-fold symmetrical double-ring^17^ (two monomers making a dimer are delineated in Fig. 1). As common for the α family of the PLP-dependent decarboxylases^17^,^25^, dimerization is required for the enzymatic activity because the active site is buried in the dimer interface (Fig. 3A,B). This interface is formed essentially by the core domains with some contribution of the CTDs. The core domain is built by the PLP-binding subdomain (PLP-SD, residues 184–417) flanked by two smaller subdomains rich in partly disordered loops – the linker region (residues 130–183) and the subdomain 4 (residues 418–563). Zooming in the variations in the PLP-SD shows that most of the structural changes concern displacements in the active site (Fig. 3C–F). The most conspicuous differences between the PLP-SDs can be linked to the pH-dependent activation of the enzymes. The resolution of the cryoEM maps does not allow modeling the position of the PLP moiety and calls for caution in detailed mechanistic interpretations in terms of individual amino acids. Therefore we restrict our analysis to secondary structure elements. In particular, transition from LdcI_i_ to LdcI-LARA involves ~3.5 Å and ~4.5 Å shifts away from the 5-fold axis in the active site α-helices spanning residues 218–232 and 246–254 respectively (Fig. 3C–E). Between these two extremes, the PLP-SDs of LdcI_a_ and LdcC are similar both in the context of the decamer (Fig. 3F) and in terms of RMSD_min_ = 0.9 Å, which probably reflects the fact that, at the optimal pH, these lysine decarboxylases have a similar enzymatic activity^26^. In addition, our earlier biochemical observation that the enzymatic activity of LdcI_a_ is unaffected by RavA binding^19^ is consistent with the relatively small changes undergone by the active site upon transition from LdcI_a_ to LdcI-LARA. Worthy of note, our previous comparison of the crystal structure of LdcI_i_ with that of the inducible arginine decarboxylase AdiA^17^ revealed high conservation of the PLP-coordinating residues and identified a patch of negatively charged residues lining the active site channel as a potential binding site for the target amino acid substrate^17^ (Figs S3 and S4 in ref. 17).

### Rearrangements of the ppGpp binding pocket upon pH-dependent enzyme activation and LARA binding

An inhibitor of the LdcI and LdcC activity, the stringent response alarmone ppGpp, is known to bind at the interface between neighboring monomers within each ring (Fig. S4). The ppGpp binding pocket is made up by residues from all domains and is located approximately 30 Å away from the PLP moiety^17^. Whereas the crystal structure of the ppGpp-LdcI_i_ was solved to 2 Å resolution, only a 4.1 Å resolution structure of the ppGpp-free LdcI_i_ could be obtained^17^. At this resolution, the apo-LdcI_i_ and ppGpp-LdcI_i_ structures (both solved at pH 8.5) appeared indistinguishable except for the presence of ppGpp^17^ (Fig. S11 in ref. 17). Thus, we speculated that inhibition of LdcI by ppGpp would be accompanied by a transduction of subtle structural changes at the level of individual amino acid side chains between the ppGpp binding pocket and the active site of the enzyme^17^. All our current cryoEM reconstructions of the lysine decarboxylases were obtained in the absence of ppGpp in order to be closer to the active state of the enzymes under study. While differences in the ppGpp binding site could indeed be visualized (Fig. S4), the level of resolution warns against speculations about their significance. The fact that interaction with RavA reduces the ppGpp affinity for LdcI^21^ despite the long distance of ~30 Å between the LARA domain binding site and the closest ppGpp binding pocket (Fig. S5) seems to favor an allosteric regulation mechanism. Interestingly, although a number of ppGpp binding residues are strictly conserved between LdcI and AdiA that also forms decamers at low pH optimal for its arginine decarboxylase activity, no ppGpp regulation of AdiA could be demonstrated^26^.

### Swinging and stretching of the CTDs upon pH-dependent LdcI activation and LARA binding

**Figure 4:**
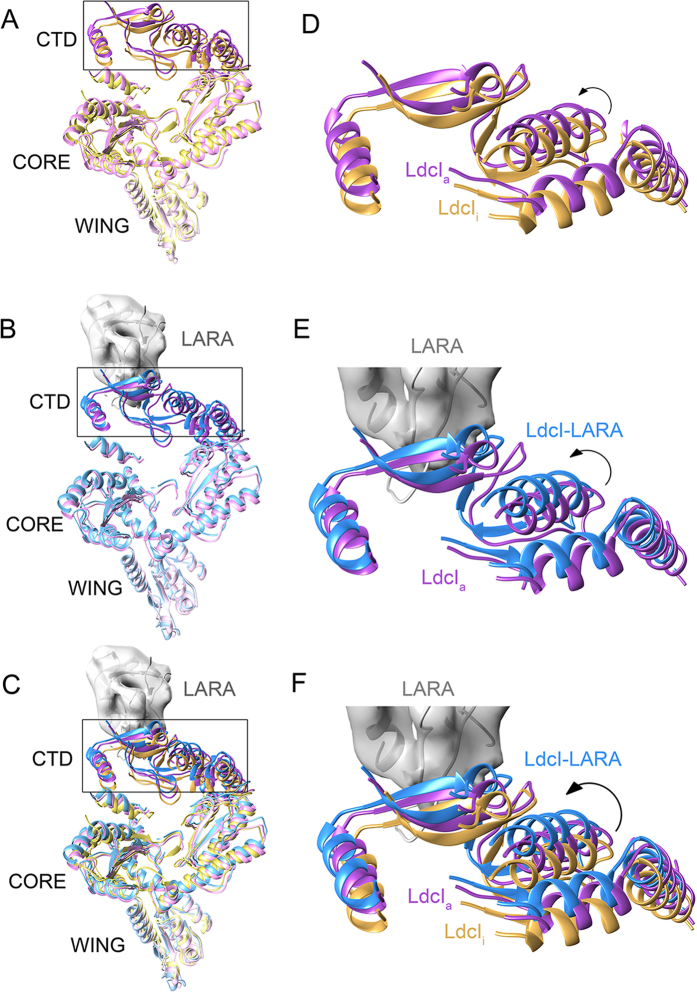
Stretching of the LdcI monomer upon pH-dependent enzyme activation and LARA binding. (**A–C**) A slice through the pseudoatomic models of the LdcI monomers extracted from the superimposed decamers (Fig. 2) The rectangle indicates the regions enlarged in (**D**–**F**). (A) compares LdcI_i_ (yellow) and LdcI_a_ (pink), (**B**) compares LdcI_a_ (pink) and LdcI-LARA (blue), and (**C**) compares LdcI_i_ (yellow), LdcI_a_ (pink) and LdcI-LARA (blue) simultaneously in order to show the progressive stretching described in the text. The cryoEM density of the LARA domain is represented as a grey surface to show the position of the binding site and the direction of the movement. (**D–F**) Inserts zooming at the CTD part in proximity of the LARA binding site. Loop regions are removed for a clearer visual comparison. An arrow indicates a swinging movement.

Inspection of the superimposed decameric structures (Figs 2 and S6) suggests a depiction of the wing domains as an anchor around which the peripheral CTDs swing. This swinging movement seems to be mediated by the core domains and is accompanied by a stretching of the whole LdcI subunits attracted by the RavA magnets. Indeed, all CTDs have very similar structures (RMSD_min_ <1 Å). Yet the superposition of the decamers lays bare a progressive movement of the CTD as a whole upon enzyme activation by pH and the binding of LARA. The LdcI_i_ monomer is the most compact, whereas LdcI_a_ and especially LdcI-LARA gradually extend their CTDs towards the LARA domain of RavA (Figs 2 and 4). These small but noticeable swinging and stretching (up to ~4 Å) may be related to the incorporation of the LdcI decamer into the LdcI-RavA cage.

### The C-terminal β-sheet of a lysine decarboxylase as a major determinant of the interaction with RavA

**Figure 5:**
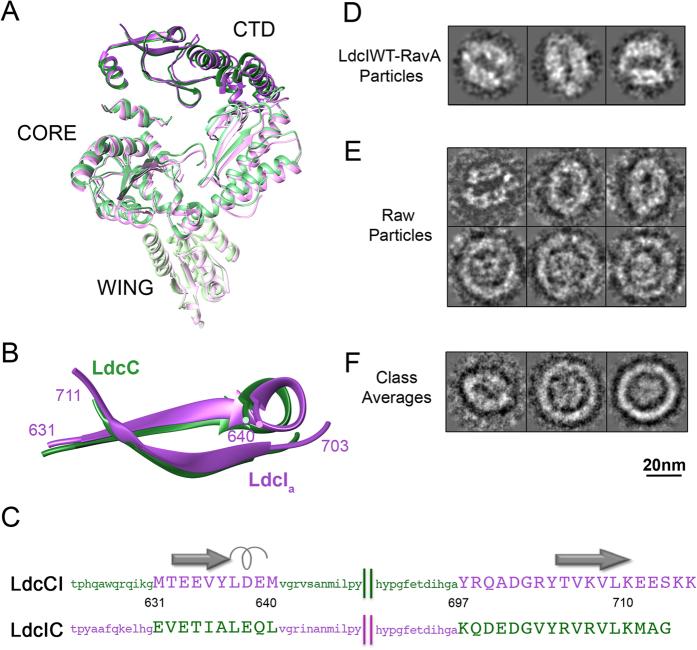
Analysis of the LdcIC and LdcCI chimeras. (**A**) A slice through the pseudoatomic models of the LdcI_a_ (purple) and LdcC (green) monomers extracted from the superimposed decamers (Fig. 2). (**B**) The C-terminal β-sheet in LdcI_a_ and LdcC enlarged from (**A**,**C**) Exchanged primary sequences (capital letters) and their immediate vicinity (lower case letters) colored as in (**A**,**B**), with the corresponding secondary structure elements and the amino acid numbering shown. (**D,E**) A gallery of negative stain EM images of (**D**) the wild type LdcI-RavA cage and (**E**) the LdcCI-RavA cage-like particles. (**F**) Some representative class averages of the LdcCI-RavA cage-like particles. Scale bar 20 nm.

In our previous contribution, based on the fit of the LdcI_i_ and the LARA crystal structures into the LdcI-LARA cryoEM density, we predicted that the LdcI-RavA interaction should involve the C-terminal two-stranded β-sheet of the LdcI^23^. Our present cryoEM maps and pseudoatomic models provide first structure-based insights into the differences between the inducible and the constitutive lysine decarboxylases. However, at the level of this structural element the two proteins are actually surpisingly similar. Therefore, we wanted to check the influence of the primary sequence of the two proteins in this region on their ability to interact with RavA. To this end, we swapped the relevant β-sheets of the two proteins and produced their chimeras, namely LdcIC (*i.e.* LdcI with the C-terminal β-sheet of LdcC) and LdcCI (*i.e.* LdcC with the C-terminal β-sheet of LdcI) (Fig. 5A–C). Both constructs could be purified and could form decamers visually indistinguishable from the wild-type proteins. As expected, binding of LdcI to RavA was completely abolished by this procedure and no LdcIC-RavA complex could be detected. On the contrary, introduction of the C-terminal β-sheet of LdcI into LdcC led to an assembly of the LdcCI-RavA complex. On the negative stain EM grid, the chimeric cages appeared less rigid than the native LdcI-RavA, which probably means that the environment of the β-sheet contributes to the efficiency of the interaction and the stability of the entire architecture (Fig. 5D–F).

### The C-terminal β-sheet of a lysine decarboxylase is a highly conserved signature allowing to distinguish between LdcI and LdcC

**Figure 6:**
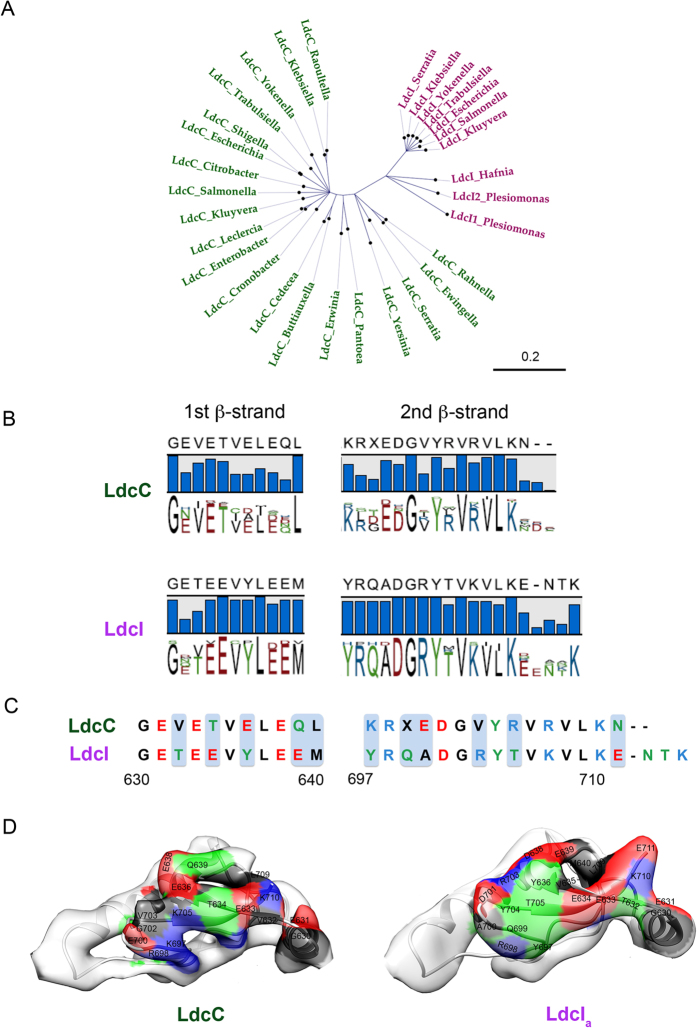
Sequence analysis of enterobacterial lysine decarboxylases. (**A**) Maximum likelihood tree with the “LdcC-like” and the “LdcI-like” groups highlighted in green and pink, respectively. Only nodes with higher values than 95% are shown (500 replicates of the original dataset, see Methods for details). Scale bar indicates the average number of substitutions per site. (**B**) Analysis of consensus “LdcI-like” and “LdcC-like” sequences around the first and second C-terminal β-strands. The height of the bars and the letters representing the amino acids reflects the degree of conservation of each particular position is in the alignment. Amino acids are colored according to a polarity color scheme with hydrophobic residues in black, hydrophilic in green, acidic in red and basic in blue. Numbering as in *E. coli*. (**C**) Signature sequences of LdcI and LdcC in the C-terminal β-sheet. Polarity differences are highlighted. (**D**) Position and nature of these differences at the surface of the respective cryoEM maps with the color code as in B. See also Fig. S7 and Tables S3 and S4.

Alignment of the primary sequences of the *E. coli* LdcI and LdcC shows that some amino acid residues of the C-terminal β-sheet are the same in the two proteins, whereas others are notably different in chemical nature. Importantly, most of the amino acid differences between the two enzymes are located in this very region. Thus, to advance beyond our experimental confirmation of the C-terminal β-sheet as a major determinant of the capacity of a particular lysine decarboxylase to form a cage with RavA, we set out to investigate whether certain residues in this β-sheet are conserved in lysine decarboxylases of different enterobacteria that have the *ravA-viaA* operon in their genome. We inspected the genetic environment of lysine decarboxylases from 22 enterobacterial species referenced in the NCBI database, corrected the gene annotation where necessary (Tables S3 and S4), and performed multiple sequence alignment coupled to a phylogenetic analysis (see Methods). This procedure yielded several unexpected and exciting results. First of all, consensus sequence for the entire lysine decarboxylase family was derived. Second, the phylogenetic analysis clearly split the lysine decarboxylases into two groups (Fig. 6A). All lysine decarboxylases predicted to be “LdcI-like” or biodegradable based on their genetic environment, as for example their organization in an operon with a gene encoding the CadB antiporter (see Methods), were found in one group, whereas all enzymes predicted as “LdcC-like” or biosynthetic partitioned into another group. Thus, consensus sequences could also be determined for each of the two groups (Figs 6B,C and S7). Inspection of these consensus sequences revealed important differences between the groups regarding charge, size and hydrophobicity of several residues precisely at the level of the C-terminal β-sheet that is responsible for the interaction with RavA (Fig. 6B–D). For example, in our previous study^23^, site-directed mutations identified Y697 as critically required for the RavA binding. Our current analysis shows that Y697 is strictly conserved in the “LdcI-like” group whereas the “LdcC-like” enzymes always have a lysine in this position; it also uncovers several other residues potentially essential for the interaction with RavA which can now be addressed by site-directed mutagenesis. The third and most remarkable finding was that exactly the same separation into “LdcI-like” and “LdcC”-like groups can be obtained based on a comparison of the C-terminal β-sheets only, without taking the rest of the primary sequence into account. Therefore the C-terminal β-sheet emerges as being a highly conserved signature sequence, sufficient to unambiguously discriminate between the “LdcI-like” and “LdcC-like” enterobacterial lysine decarboxylases independently of any other information (Figs 6 and S7). Our structures show that this motif is not involved in the enzymatic activity or the oligomeric state of the proteins. Thus, enterobacteria identified here (Fig. 6, Table S4) appear to exert evolutionary pressure on the biodegradative lysine decarboxylase towards the RavA binding. One of the elucidated roles of the LdcI-RavA cage is to maintain LdcI activity under conditions of enterobacterial starvation by preventing LdcI inhibition by the stringent response alarmone ppGpp^21^. Furthermore, the recently documented interaction of both LdcI^27^ and RavA^22^ with specific subunits of the respiratory complex I, together with the unanticipated link between RavA and maturation of numerous iron-sulfur proteins, tend to suggest an additional intriguing function for this 3.5 MDa assembly. The conformational rearrangements of LdcI upon enzyme activation and RavA binding revealed in this work, and our amazing finding that the molecular determinant of the LdcI-RavA interaction is the one that straightforwardly determines if a particular enterobacterial lysine decarboxylase belongs to “LdcI-like” or “LdcC-like” proteins, should give a new impetus to functional studies of the unique LdcI-RavA cage. Besides, the structures and the pseudoatomic models of the active ppGpp-free states of both the biodegradative and the biosynthetic *E. coli* lysine decarboxylases offer an additional tool for analysis of their role in UPEC infectivity. Together with the apo-LdcI and ppGpp-LdcI_i_ crystal structures, our cryoEM reconstructions provide a structural framework for future studies of structure-function relationships of lysine decarboxylases from other enterobacteria and even of their homologues outside *Enterobacteriaceae*. For example, the lysine decarboxylase of *Eikenella corrodens* is thought to play a major role in the periodontal disease and its inhibitors were shown to retard gingivitis development^28^,^29^,^30^. Finally, cadaverine being an important platform chemical for the production of industrial polymers such as nylon, structural information is valuable for optimisation of bacterial lysine decarboxylases used for its production in biotechnology^31^,^32^,^33^.

## Methods

### Protein expression and purification

LdcI and LdcC were expressed and purified as described^19^,^23^,^26^ from an *E. coli* strain that cannot produce ppGpp (MG1655 Δ*relA* Δ*spoT* strain). LdcI was stored in 20 mM Tris-HCl, 100 mM NaCl, 1 mM DTT, 0.1 mM PLP, pH 6.8 (buffer A) and LdcC in 20 mM Tris-HCl, 100 mM NaCl, 1 mM DTT, 0.1 mM PLP, pH 7.5 (buffer B).

Chimeric LdcIC and LdcCI were constructed, expressed and purified as follows. The chimeras were designed by exchange, between LdcI and LdcC, of residues from 631 to 640 and from 697 to the C-terminus, corresponding to the regions around the two strands of the C-terminal β-sheet (Fig. 5B,C), while leaving the rest of the sequence unaltered. The synthetic *ldcIC* and *ldcCI* genes (2148 bp and 2154 bp respectively), provided within a pUC57 vector (GenScript) were subcloned into pET-TEV vector based on pET-28a (Invitrogen) containing an N-terminal TEV-cleavable 6x-His-Tag. Proteins were expressed in Rosetta 2 (DE3) cells (Novagen) in LB medium supplemented with kanamycin and chloramphenicol at 37 °C, upon induction with 0.5 mM IPTG at 18 °C. Cells were harvested by centrifugation, the pellet resuspended in a 50 mM Tris-HCl, 150 mM NaCl, pH 8 buffer supplemented with Complete EDTA free (Roche) and 0.1 mM PMSF (Sigma), and disrupted by sonication at 4 °C. After centrifugation at 75000 g at 4 °C for 20 min, the supernatant was loaded on a Ni-NTA column. The eluted protein-containing fractions were pooled and the His-Tag removed by incubation with the TEV protease at 1/100 ratio and an extensive dialysis in a 50 mM Tris-HCl, 150 mM NaCl, 1 mM DTT, 5 mM EDTA, pH 8 buffer. After a second dialysis in a 50 mM Tris-HCl, 150 mM NaCl, pH 8 buffer supplemented with 10 mM imidazole, the sample was loaded on a Ni-NTA column in the same buffer, which allowed to separate the TEV protease and LdcCI/LdcIC. Finally, the pure proteins were obtained by size exclusion chromatography on a Superdex-S200 column in buffer A.

### LdcI_a_ -cryoEM data collection and 3D reconstruction

LdcI was prepared at 2 mg/ml in a buffer containing 25 mM MES, 100 mM NaCl, 0.2 mM PLP, 1 mM DTT, pH 6.2. 3 μl of sample were applied to glow-discharged quantifoil grids 300 mesh 2/1 (Quantifoil Micro Tools GmbH, Germany), excess solution was blotted during 2.5 s with a Vitrobot (FEI) and the grid frozen in liquid ethane^34^. Data collection was performed on a FEI Polara microscope operated at 300 kV under low dose conditions. Micrographs were recorded on Kodak SO-163 film at 59,000 magnification, with defocus ranging from 0.6 to 4.9 μm. Films were digitized on a Zeiss scanner (Photoscan) at a step size of 7 μm giving a pixel size of 1.186 Å. The contrast transfer function (CTF) for each micrograph was determined with CTFFIND3^35^.

Initially ~2500 particles of 256 × 256 pixels were extracted manually, binned 4 times and subjected to one round of multivariate statistical analysis and classification using IMAGIC^36^. Representative class averages corresponding to projections in different orientations were used as input for an ab-initio 3D reconstruction by RICOserver (*rico.ibs.fr/)*^37^. The resulting 3D reconstruction was refined using RELION^38^, which yielded an 18 Å resolution map. Using projections of this model as a template, particles of size 256 × 256 pixels were semi-automatically selected from all the micrographs using the Fast Projection Matching (FPM) algorithm^39^. The resulting dataset of ~46000 particles was processed in RELION with the previous map as an initial model and with a full CTF correction after the first peak. The final map comprised 44207 particles with a resolution of 7.4 Å as per the gold-standard FSC = 0.143 criterion^40^. It was sharpened with EMBfactor^41^ using calculated B-factor of −350 Å^2^ and masked with a soft mask to obtain a final map with a resolution of 6.1 Å (Fig. S3, Table S1).

### LdcC - cryoEM data collection and 3D reconstruction

LdcC was prepared at 2 mg/ml in a buffer containing 25 mM HEPES, 100 mM NaCl, 0.2 mM PLP, 1 mM DTT, pH 7.2. Grids were prepared and sample imaged as LdcI_a_. Data were processed essentially as LdcI_a_ described above. Briefly, an initial ~20 Å resolution model was generated by angular reconstitution after manual picking of circa 3000 particles from the first micrographs, filtered to 60 Å resolution, refined with RELION and used for a semi-automatic selection with FPM. The dataset was processed in RELION with a full CTF correction to yield a final reconstruction comprising 61000 particles. The map was sharpened with Relion postprocessing, using a soft mask and automated B-factor estimation (−690 Å^2^), yielding a map at 5.5 Å resolution (Fig. S1, Table S1).

### LdcI-LARA - 3D reconstruction

In our first study^23^, the dataset was processed in SPIDER and the CTF correction involved a simple phase-flipping. Therefore, for consistency with the present work, we revisited the dataset and processed it in RELION with a full CTF correction after the first peak. It was sharpened with EMBfactor^41^ using calculated B-factor of −350 Å^2^ and masked with a soft mask to obtain a final map with a resolution of 6.2 Å (Fig. S2). This reconstruction is of a slightly better quality in terms of the continuity of the internal density. Therefore we used this improved map for fitting of the atomic model and further analysis (Fig. S2, Table S1).

### Additional image processing

As a crosscheck, each data set was also refined either from other initial models: a “featureless donut” with approximate dimensions of the decamer, and low pass-filtered reconstructions from the two other data sets (i.e. the LdcC reconstruction was used as a model for the LdcI_a_ and LdcI-LARA data sets, etc). All refinements converged to the same solutions independently of the starting model. Local resolution of all maps was determined with ResMap^42^.

### LdcCI and LdcIC chimeras —negative stain EM and 2D image analysis

0.4 mg/ml of RavA (in a 20 mM Tris-HCl, 500 mM NaCl, 10 mM MgCl_2_, 1 mM DTT, 5% glycerol, pH 6.8 buffer) was mixed with 0.3 mg/ml of either LdcI, LdcC, LdcCI or LdcIC in the presence of 2 mM ADP and 10 mM MgCl_2_ in a buffer containing 20 mM Hepes and 150 mM NaCl at pH 7.4. After 10 minutes incubation at room temperature, 3 μl of mixture were applied to the clear side of the carbon on a carbon-mica interface and negatively stained with 2% uranyl acetate. Images were recorded with a JEOL 1200 EX II microscope at 100 kV at a nominal magnification of 15000 on a CCD camera yielding a pixel size of 4.667 Å. No complexes between RavA and LdcC or LdcIC could be observed, whereas the LdcCI-RavA preparation manifested cage-like particles similar to the previously published LdcI-RavA^19^, but also unbound RavA and LdcCI, which implies that the affinity of RavA to the LdcCI chimera is lower than its affinity to the native LdcI. 1260 particles of 96 × 96 pixels were extracted interactively from several micrographs. 2D centering, multivariate statistical analysis and classification were performed using IMAGIC^36^. Class-averages similar to the cage-like LdcI-RavA complex were used as references for multi-reference alignment followed by multivariate statistical analysis and classification.

### Fitting of atomic models into cryoEM maps

A homology model of LdcC was obtained using the atomic coordinates of the LdcI monomer (3N75) as the template in SWISS-MODEL server^43^. The RMSD between the template and the resulting model was 0.26 Å. The LdcC model was then fitted as a rigid body into the LdcC cryoEM map using the fit-in-map module of UCSF Chimera^44^. This rigid fit indicated movements of several parts of the protein. Therefore, the density corresponding to one LdcC monomer was extracted and flexible fitting was performed using IMODFIT^45^ at 8 Å resolution. This monomeric model was then docked into the decameric cryoEM map with URO^46^ and its graphical version VEDA^47^ that use symmetry information for fitting in Fourier space. The Cα RMSD_min_ between the initial model of the LdcC monomer and the final IMODFIT LdcC model is 1.2 Å. In the case of LdcI_a_, the density corresponding to an individual monomer was extracted and the fit performed similarly to the one described above, with the final model of the decameric particle obtained with URO and VEDA. The Cα RMSD_min_ between the LdcI_i_ monomer and the final IMODFIT model is 1.4 Å. For LdcI-LARA, the density accounting for one LdcI monomer bound to a LARA domain was extracted and further separated into individual densities corresponding to LdcI and to LARA. The fit of LdcI was performed as for LdcC and LdcI_a_, while the crystal structure of LARA was docked into the monomeric LdcI-LARA map as a rigid body using SITUS. The resulting pseudoatomic models were used to create the final model of the LdcI-LARA decamer with URO and VEDA. The Cα RMSD_min_ between the LdcI_i_ monomer and the final IMODFIT model is 1.4 Å. A brief summary of relevant parameters is provided in Table S1.

### Sequence analysis

Out of a non-exhaustive list of 50 species of *Enterobacteriaceae* (Table S3), 22 were found to contain genes annotated as *ldcI* or *ldcC* and containing the *ravA-viaA* operon (Table S4). An analysis using MUSCLE^48^ with default parameters showed that these genes share more than 65% identity. To verify annotation of these genes, we compared their genetic environment with that of *E. coli ldcI* and *ldcC.* Indeed, in *E. coli* the *ldcI* gene is in operon with the c*adB* gene encoding a lysine-cadaverine antiporter, whereas the *ldcC* gene is present between the *accA* gene (encoding an acetyl-CoA carboxylase alpha subunit carboxyltransferase) and the *yaeR* gene (coding for an unknown protein belonging to the family of Glyoxalase/Dioxygenase/Bleomycin resistance proteins). Compared with this genetic environment, the annotation of several *ldcI* and *ldcC* genes in enterobacteria was found to be inconsistent (Table S4). For example, several strains contain genes annotated as *ldcC* in the genetic background of *ldcI* and *vice versa*, as in the case of *Salmonella enterica* and *Trabulsiella guamensi.* Furthermore, the gene with an “*ldcC*-like” environment was found to be annotated as *cadA* in particular strains of *Citrobacter freundii, Cronobacter sakazakii, Enterobacter cloacae* subsp. *Cloaca, Erwinia amylovora, Pantoea agglomerans, Rahnella aquatilis, Shigella dysenteriae*, and *Yersinia enterocolitica* subsp. *enterocolitica,* whereas in *Hafnia alvei, Kluyvera ascorbata,* and *Serratia marcescens* subsp. *marcescens,* the gene with an “*ldcI*-like” environment was found to be annotated as *ldcC*. In addition, as far as the genetic environment is concerned, *Plesiomonas* appears to have two *ldc* genes with the organization of the *E. coli ldcI* (operon *cadA-cadB*). Consequently, we corrected for gene annotation consistent with the genetic environment and made multiple sequence alignments using version 8.0.1 of the CLC Genomics Workbench software. A phylogenetic tree was generated based on Maximum Likelihood and following the Neighbor-Joining method with the WAG protein substitution model^49^. The reliability of the generated phylogenetic tree was assessed by bootstrap analysis. The presented unrooted phylogenetic tree shows the nodes that are reliable over 95% (Fig. 6A). Remarkably, the multiple sequence alignment and the resulting phylogenetic tree clearly grouped together all sequences annotated as *ldcI* on the one side, and all sequences annotated as *ldcC* on the other side. Thus, we conclude that all modifications in gene annotations that we introduced for the sake of consistency with the genetic environment are perfectly corroborated by the multiple sequence alignment and the phylogenetic analysis. Since the regulation of the lysine decarboxylase gene (i.e. inducible or constitutive) cannot be assessed by this analysis, we call the resulting groups “*ldcI*-like” and “*ldcC*-like” as referred to the *E. coli* enzymes, and make a parallel between the membership in a given group and the ability of the protein to form a cage complex with RavA.

## Additional Information

**Accession codes:** CryoEM maps and corresponding fitted atomic structures (main chain atoms) have been deposited in the Electron Microscopy Data Bank and Protein Data Bank, respectively, with accession codes EMD-3205 and 5FKZ for LdcC, EMD-3204 and 5FKX for LdcI_a_ and EMD-3206 and 5FL2 for LdcI-LARA.

**How to cite this article**: Kandiah, E. *et al.* Structural insights into the *Escherichia coli* lysine decarboxylases and molecular determinants of interaction with the AAA+ ATPase RavA. *Sci. Rep.* **6**, 24601; doi: 10.1038/srep24601 (2016).

## Supplementary Material

Supplementary Information

Supplementary Movie 1
