# Supplementary material for: Structural insights into the Escherichia coli lysine decarboxylases and molecular determinants of interaction with the AAA+ ATPase RavA
Source: Sci Rep. 2016 Apr 15;6:24601. doi: 10.1038/srep24601 (PMC4832331; doi:10.1038/srep24601)
Supplement: Supplementary Information [file srep24601-s1.pdf]

Structural insights into the *Escherichia coli* lysine decarboxylases and molecular determinants of interaction with the AAA+ ATPase RavA

Eaazhisai Kandiah[1,2,3], Diego Carriel[1,2,3,4,5,6,7], Julien Perard[6,7,8], Hélène Malet[1,2,3], Maria Bacia[1,2,3], Kaiyin Liu[9], Sze W. S. Chan[9], Walid A. Houry[9], Sandrine Ollagnier de Choudens[6,7,8], Sylvie Elsen[4,5,6,7] and Irina Gutsche[1,2,3,*]

[1]University Grenoble Alpes, IBS, F-38044 Grenoble, France
[2]CNRS, IBS, F-38044 Grenoble, France
[3]CEA, IBS, F-38044 Grenoble, France
[4]INSERM, Biologie du Cancer et de l'Infection (UMR-S1036), F-38054 Grenoble, France
[5]Centre National de la Recherche Scientifique (CNRS), ERL5261
[6]University Grenoble Alpes, BIG, Grenoble, F-38041, France
[7]CEA, BIG, Grenoble, France
[8]CNRS, LCMB, BIG, Grenoble, France
[9]Department of Biochemistry, University of Toronto, Toronto, Ontario M5S 1A8, Canada

* Corresponding author
Email: irina.gutsche@ibs.fr
Phone: + 33 4 57 42 87 66

Supplementary figure legends

Figure S1. Brief summary of the LdcC image analysis. (A) CryoEM micrograph. (B) Gold-standard FSC curves calculated for unmasked (dark green) and soft shaped masked (light green) maps, indicating the resolution of 6.0 Å and 5.5 Å respectively according to FSC=0.143 criterion. (C) Local resolution calculation from ResMap. A segmented monomer is shown in two different orientations.

Supplementary Fig. S1

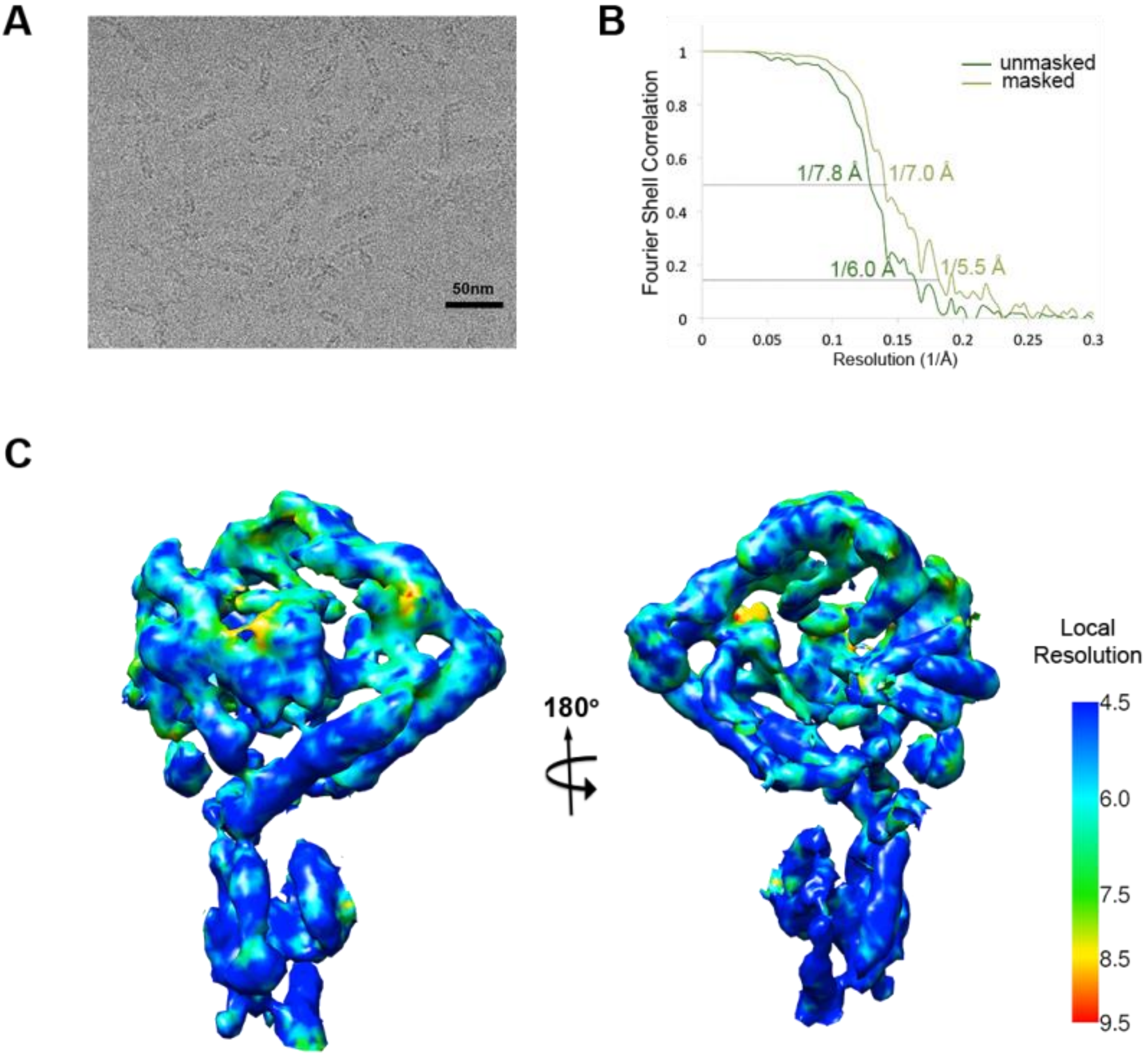

Figure S2. Brief summary of the LdcI image analysis. (A) CryoEM micrograph. (B) Gold-standard FSC curves calculated for unmasked (dark purple) and soft shaped masked (light purple) maps, indicating the resolution of 7.4 Å and 6.1 Å respectively according to FSC=0.143 criterion. (C) Local resolution calculation from ResMap. A segmented monomer is shown in two different orientations.

Supplementary Fig. S2

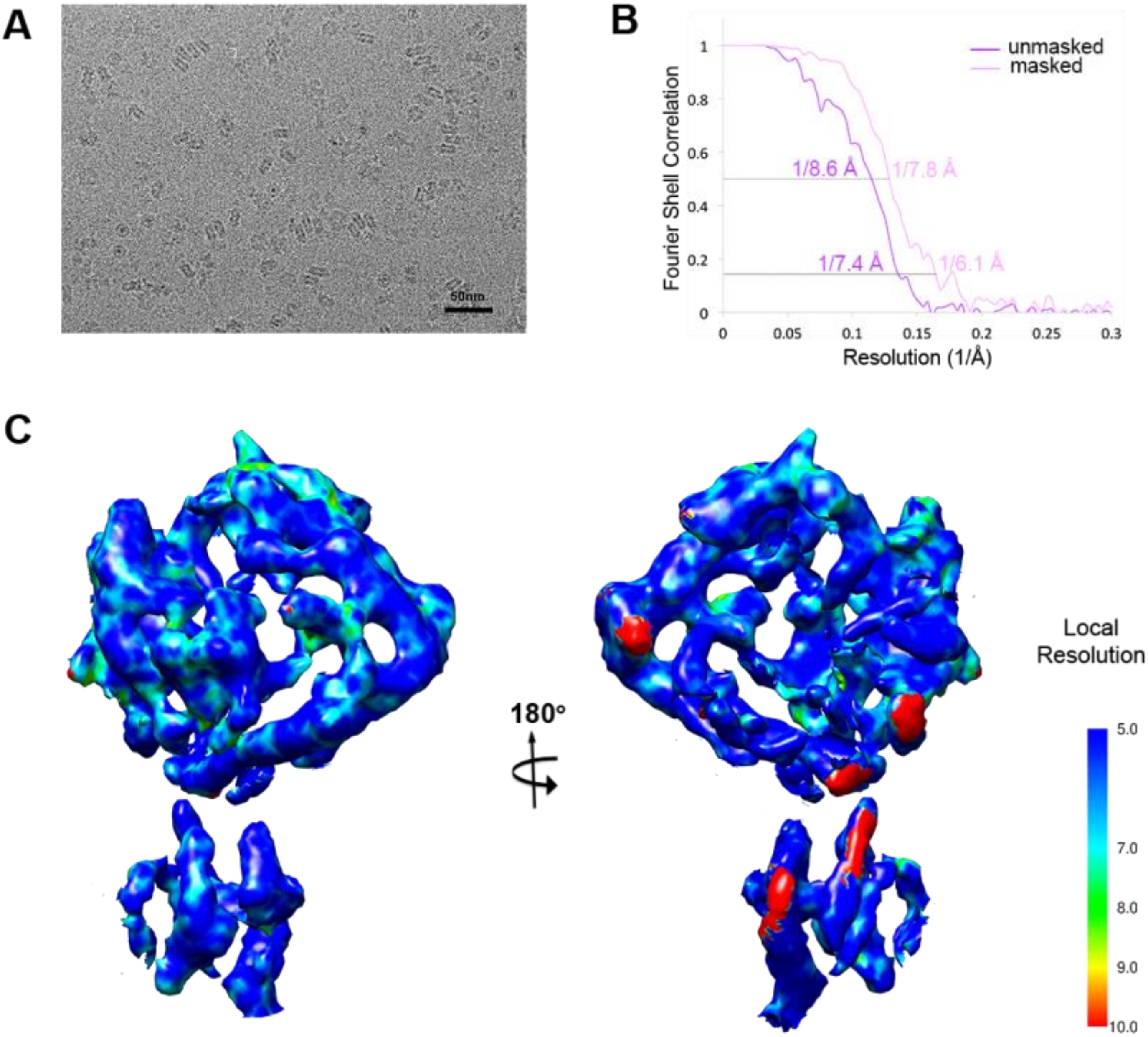

Figure S3. Comparison between the previous and the current LdcI-LARA reconstructions. (A) Gold-standard FSC curves calculated for unmasked (dark green) and soft shaped masked (light green) current LdcI-LARA maps, indicating the resolution of 6.2 Å and 7.8 Å respectively according to FSC=0.143 criterion. (B) Local resolution calculation from ResMap. A segmented LdcI-LARA monomer is shown in two different orientations. (C, D) Slightly improved connectivities in several regions of the current LdcI-LARA map in comparison to the previous one (Malet et al., 2014). (C) Slices through the decamer density maps. (D) Slices through isosurface representation with the pseudoatomic model. Arrows indicate areas for comparison.

Supplementary Fig. S3

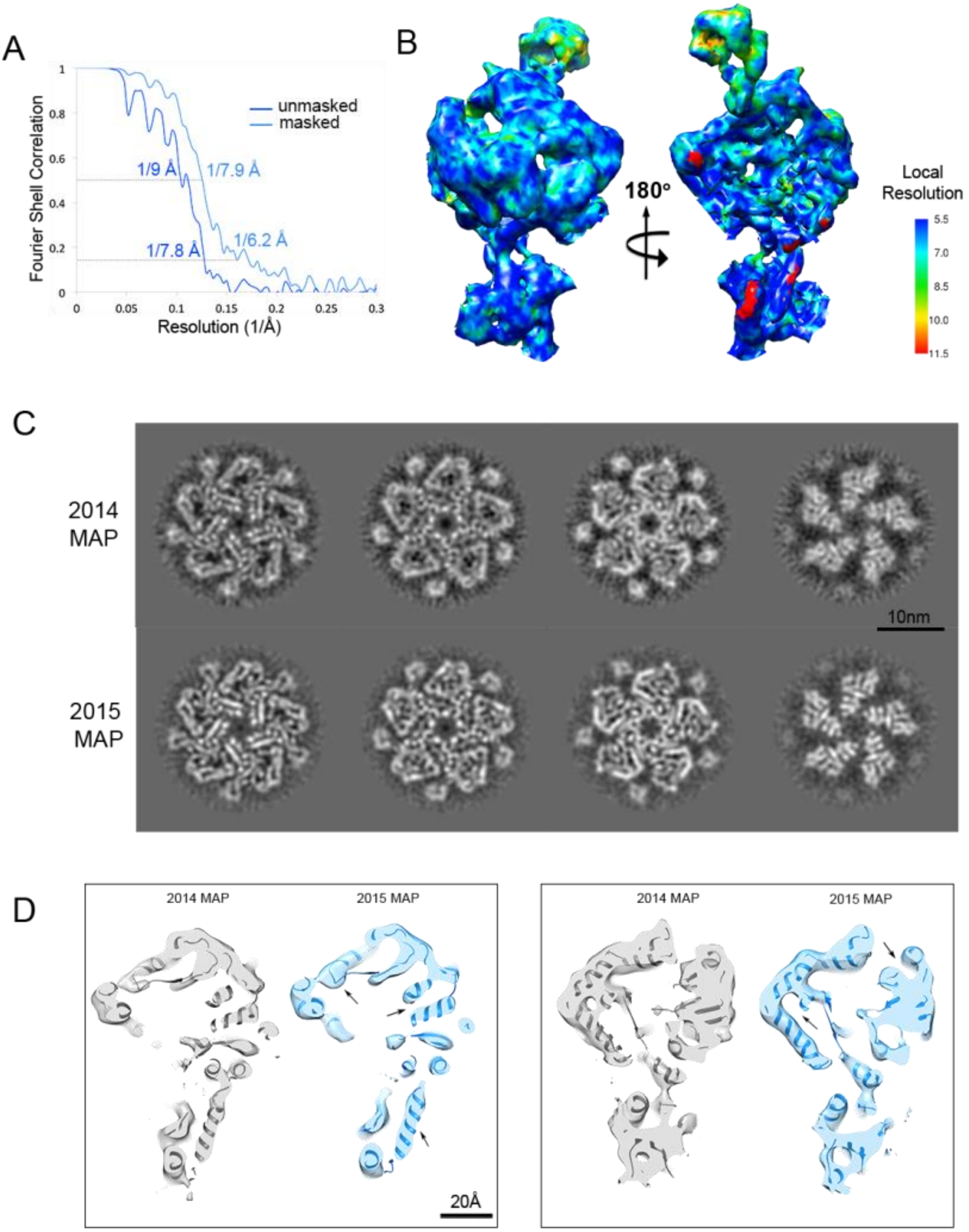

Figure S4. Conformational rearrangements in the ppGpp binding site. (A) $LdcI_i$ crystal structure, with one ring represented as a grey surface and the second as a cartoon. One monomer is delineated. Contrary to the PLP moiety that binds at the 2-fold symmetric dimer interface between the two rings (Fig. 3), ppGpp (in red) binds between each two subunits of the same ring. (B) Close-up view of the ppGpp binding pocket in the apo-$LdcI_i$ crystal structure (PDB ID 3Q16, beige) and in the ppGpp-$LdcI_i$ (PDB ID 3N75, yellow) solved at 4.1 Å and 2 Å resolution respectively (Kanjee et al, EMBO j 2011). This panel shows that at the level of the secondary structure elements both structures are virtually identical except for the presence of ppGpp. (C-F) Close-up views of the ppGpp binding pocket. The ppGpp moiety in red is from the $LdcI_i$ crystal structure, while all cryoEM maps were obtained in a non-inhibited ppGpp-free state. The intersubunit interface is shown as a dashed line. (C) compares $LdcI_i$ (yellow) and $LdcI_a$ (pink), (D) compares $LdcI_a$ (pink) and LdcI-LARA (blue), and (E) compares $LdcI_i$ (yellow), $LdcI_a$ (pink) and LdcI-LARA (blue) simultaneously. (F) compares $LdcI_a$ and LdcC. Colours as in the other figures.

Supplementary Fig. S4

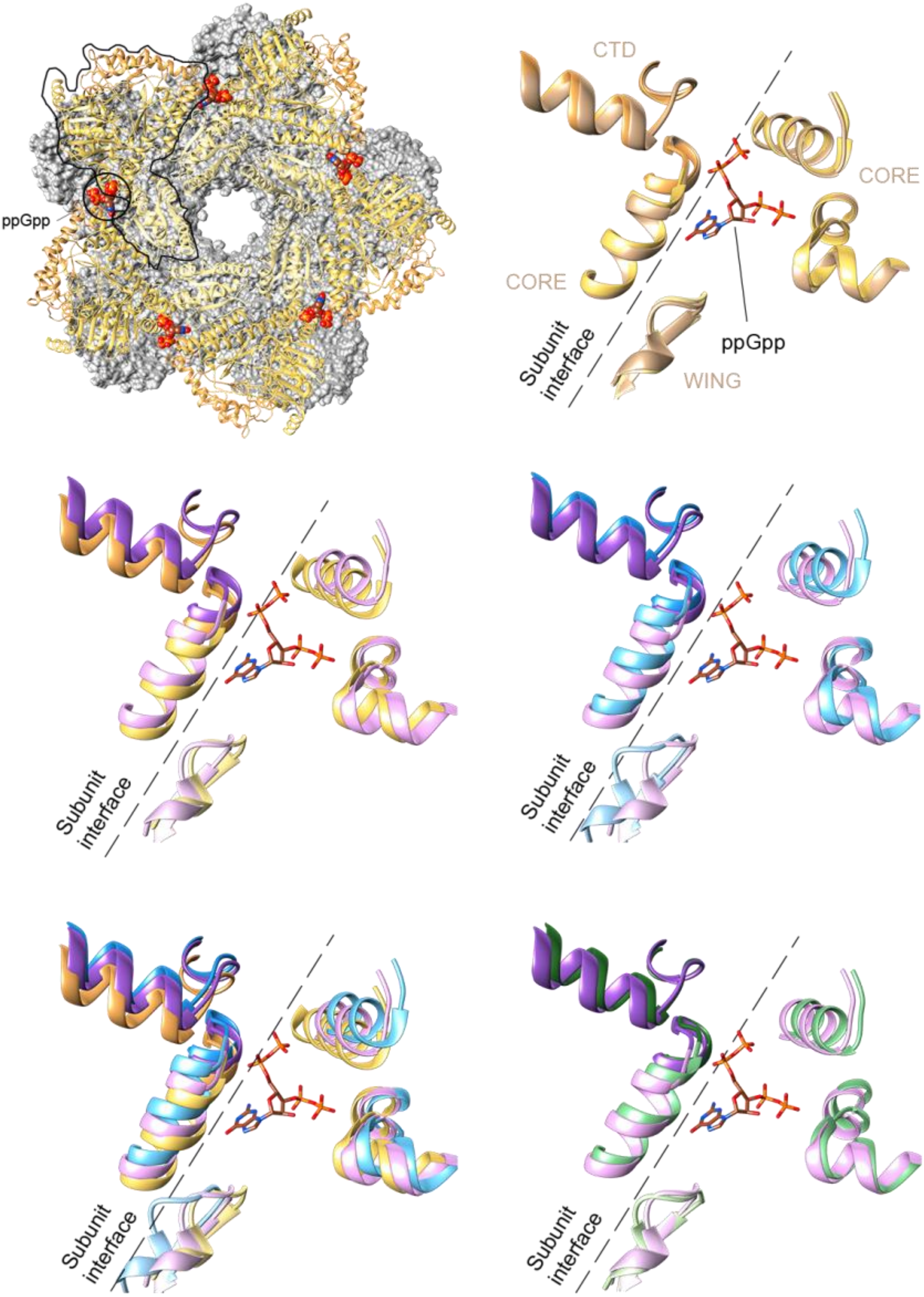

Figure S5. Distances between PLP, ppGpp and the LARA binding site (Y697). (A) $LdcI_i$ crystal structure, with one ring represented as a grey surface and the second as a cartoon with each monomer in a different color. PLP is shown in purple, ppGpp in orange. Subunits 1 and 2 belong to the same dimer. Two LARA domains from the LdcI-LARA structure are shown in order to remind where the LARA binding site is located. The LARA domain bound to subunit 1 is termed LARA1 and the LARA domain bound to the subunit 2 is termed LARA2. (B) Distances between the LdcI residue Y697 (shown to be critically required for the LARA binding and described in the main text) on subunits 1 and 2, the PLP of the Subunit 1 and the two neighboring ppGpp molecules (called ppGpp and ppGpp") are shown on top of a schematic representation. As explained in the main text, the length of these distances seems to suggest an existence of an allosteric regulation of the LdcI activity by ppGpp and RavA.

Supplementary Fig. S5

A

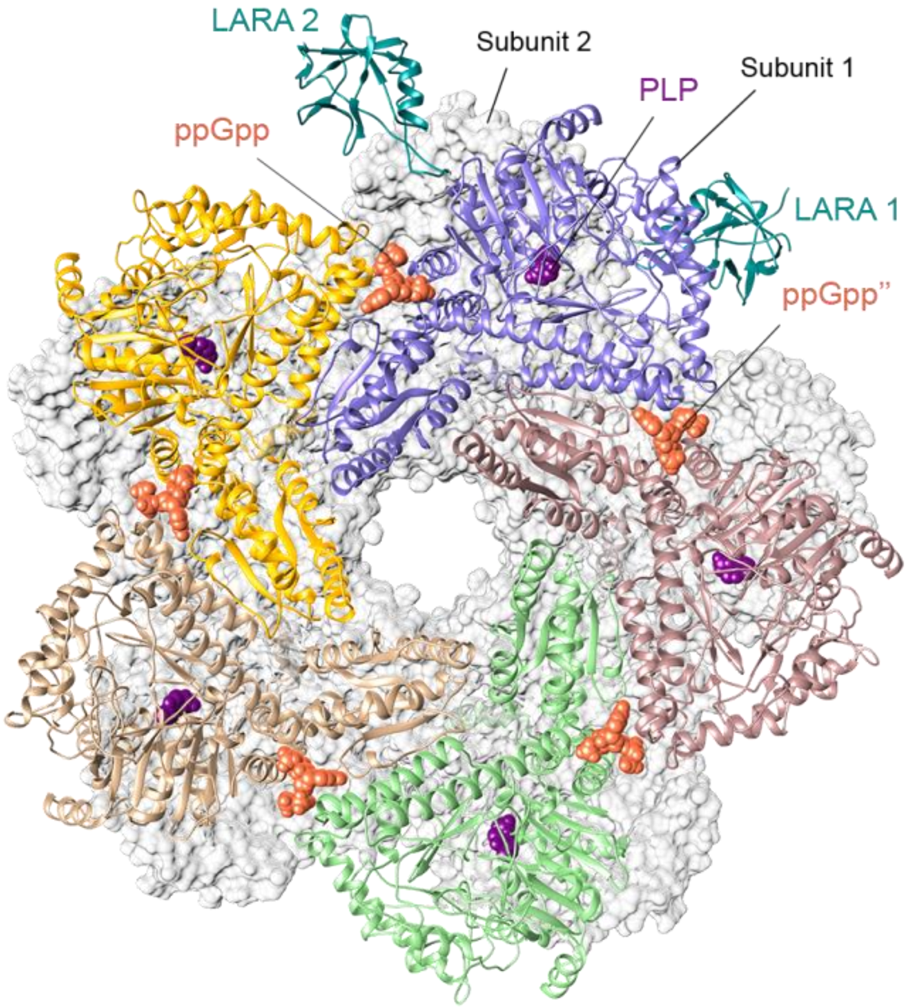

B

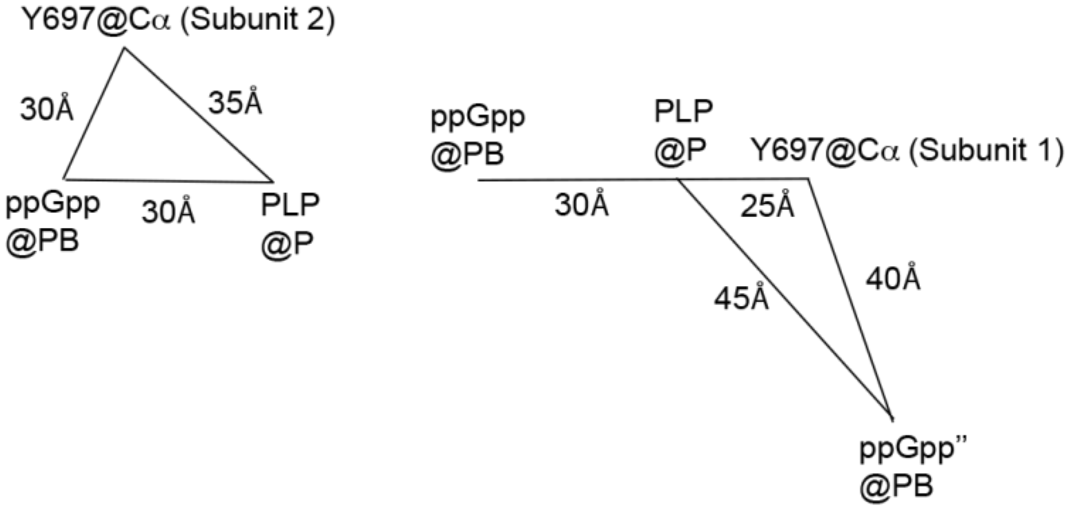

Figure S6. Comparison between the cryoEM maps of the LdcI alone and incorporated into the LdcI-LARA complex. For the LdcI-LARA complex, only the LdcI part is shown in order to facilitate direct visual comparison of the two maps. As in Fig. 1, LdcI is represented in shades of pink and the LdcI part of the LdcI-LARA complex in shades of blue (the lightest shade for the wing domains, the intermediate shade for the core domains and the darkest shade for the CTDs). For clarity, only parts of the structure are shown. (A) One ring of the decamer viewed from the top. (B) A slice of the side view of the double ring. Scale bar 50 Å.

Supplementary Fig. S6

A

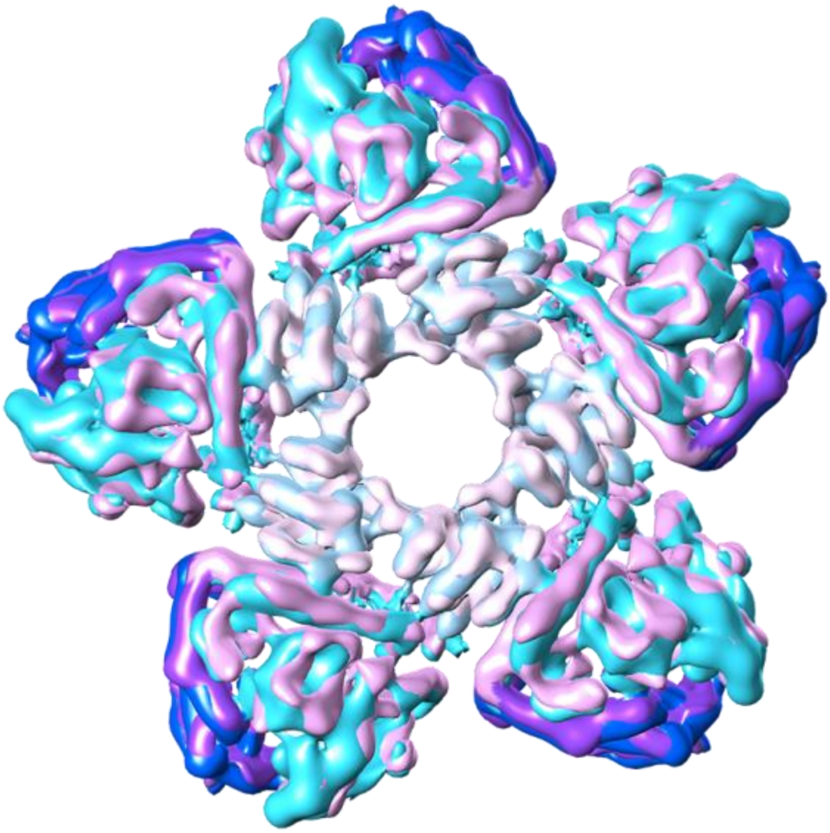

B

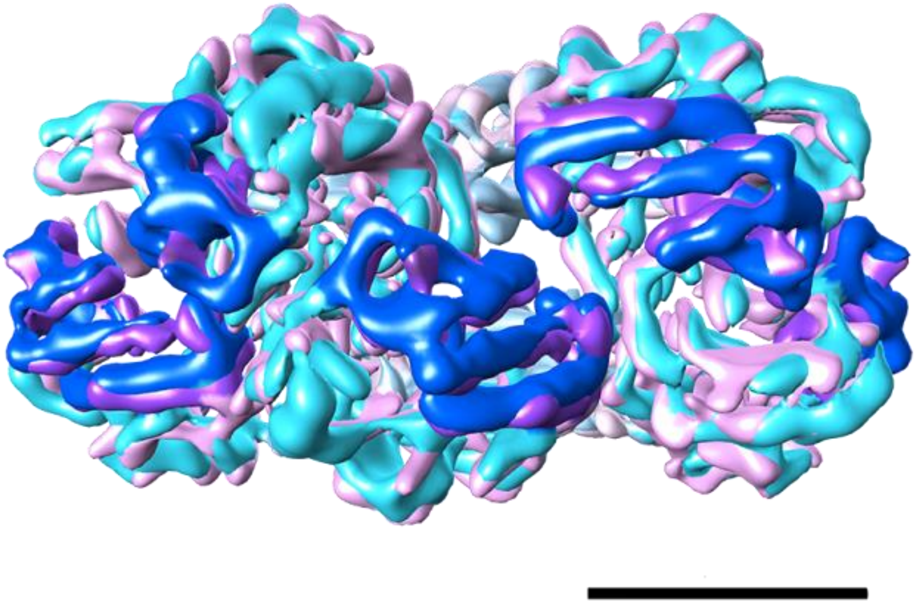

Figure S7. Alignment of the region around the first and second C-terminal β-strands for the 22 enterobacterial lysine decarboxylase sequences examined. The resulting clear subdivision in "LdcI-like" and "LdcC-like" groups and the derived consensus sequences for each group are shown. Numbering as in *E. coli*.

Supplementary Fig. S7

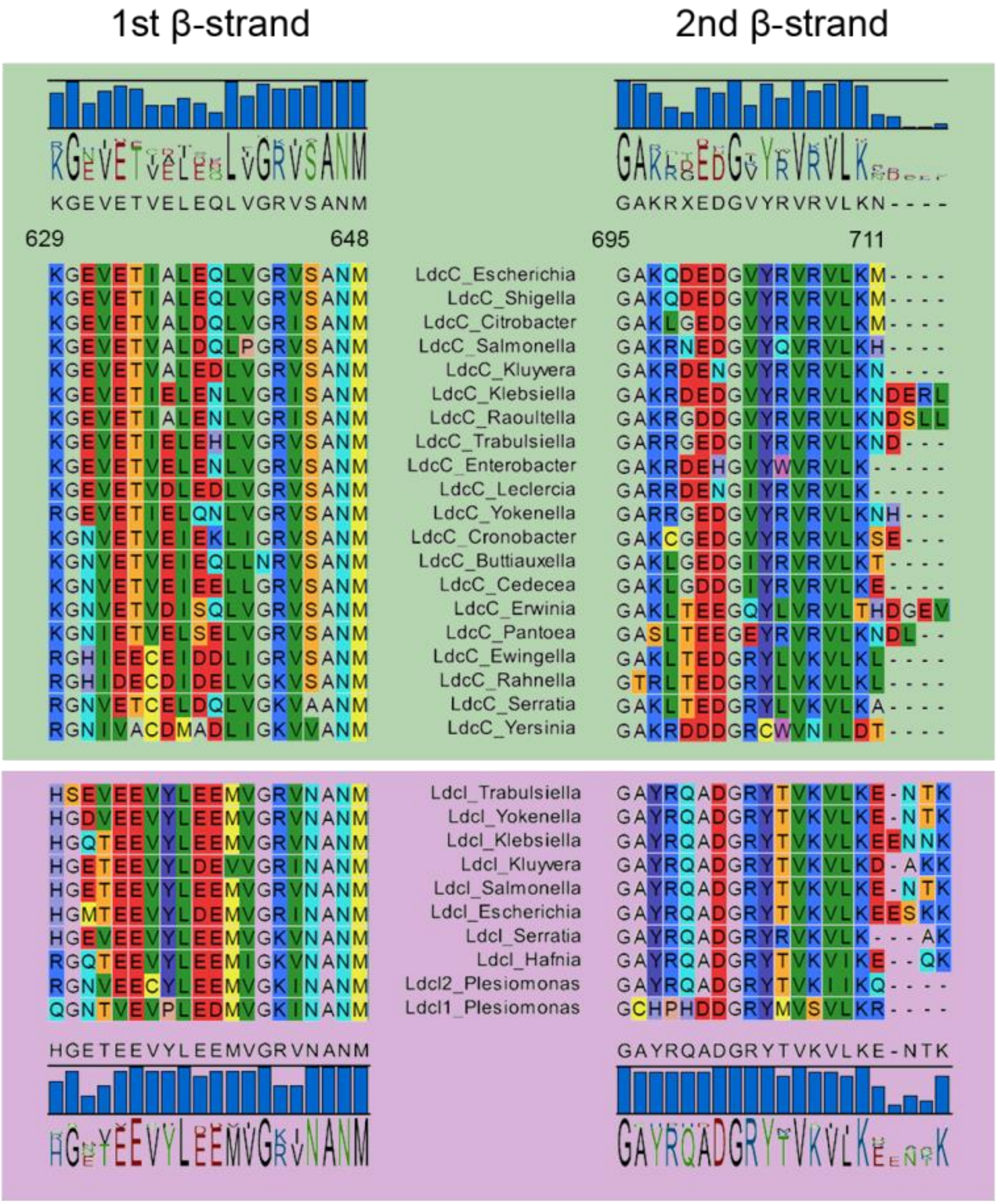

Table S1. Brief summary of image analysis and pseudoatomic model building.

Supplementary Table S1

Protein Sample	LdcC	$LdcI_a$	LdcI-LARA
Defocus Range (µm)	0.5 – 4.2	0.6 – 4.9	1.5 – 2.7
Final # of particles	58296	44207	23540
CTF correction	Full-CTF	Full-CTF after 1st peak	Full-CTF after 1st peak
Resolution (Å)	5.5	6.1	6.2
B-factor ($Å^2$)	-690	-350	-350
MolProbity *			
Clash score	0.35	1.06	2.11
Ramachandran outliers (%)	0.14	0.28	0.42
Overall score	0.83	1.10	1.27

* Only main chain atoms were included

Table S2. RMSDs between either the entire monomers (A) or individual domains (B for wing, C for core and D for CTD) of $LdcI_i$, $LdcI_a$, LdcI-LARA and LdcC calculated directly from superimposed decamers. Only Cα atoms are used for calculations.

Supplementary Table S2

A

**Monomer**	**$LdcI_i$**	**$LdcI_a$**	**LdcI-LARA**	**LdcC**
**$LdcI_i$**				
**$LdcI_a$**	2.1			
**LdcI-LARA**	3.4	2.0		
**LdcC**	2.6	1.7	1.8	

B

**Wing**	**$LdcI_i$**	**$LdcI_a$**	**LdcI-LARA**	**LdcC**
**$LdcI_i$**				
**$LdcI_a$**	1.4			
**LdcI-LARA**	2.0	1.5		
**LdcC**	1.7	1.6	1.4	

C

**Core**	**$LdcI_i$**	**$LdcI_a$**	**LdcI-LARA**	**LdcC**
**$LdcI_i$**				
**$LdcI_a$**	2.1			
**LdcI-LARA**	3.5	2.0		
**LdcC**	2.5	1.7	1.7	

D

**CTD**	**$LdcI_i$**	**$LdcI_a$**	**LdcI-LARA**	**LdcC**
**$LdcI_i$**				
**$LdcI_a$**	2.4			
**LdcI-LARA**	4.2	2.4		
**LdcC**	3.3	1.9	2.1	

Table S3. Presence of an *ldc* gene in the 50 enterobacterial species analysed

Supplementary Table S3

N°	Organism	*ldc* gene	*ravAviaA genes*
1	*Arsenophonus nasoniae*	NO	NO
2	*Biostraticola tofi*	NO	NO
3	*Brenneria salicis*	NO	NO
4	*Budvicia aquatica*	NO	YES
5	*Buchnera aphidicola*	NO	YES
6	*Buttiauxella agrestis*	YES	YES
7	*Cedecea davisae*	YES	YES
8	*Citrobacter freundii*	YES	YES
9	*Cosenzaea myxofaciens*	NO	NO
10	*Cronobacter sakazakii*	YES	YES
11	*Dickeya chrysanthemi*	NO	YES
12	*Edwardsiella tarda*	YES	NO
13	*Enterobacter cloacae subsp. cloacae*	YES	YES
14	*Erwinia amylovora*	YES	YES
15	*Escherichia coli*	YES	YES
16	*Ewingella americana*	YES	YES
17	*Gibbsiella quercinecans*	NO	NO
18	*Hafnia alvei*	YES	YES
19	*Klebsiella pneumoniae subsp. pneumoniae*	YES	YES
20	*Kluyvera ascorbata*	YES	YES
21	*Leclercia adecarboxylata*	YES	YES
22	*Leminorella grimontii*	NO	YES
23	*Lonsdalea quercina subsp. quercina*	NO	YES
24	*Moellerella wisconsensis*	NO	YES
25	*Morganella morganii subsp. morganii*	NO	YES
26	*Pantoea agglomerans*	YES	YES
27	*Pectobacterium carotovorum subsp. carotovorum*	NO	YES
28	*Photorhabdus luminescens subsp. luminescens*	NO	NO
29	*Plesiomonas shigelloides*	YES	YES
30	*Pragia fontium*	NO	YES
31	*Proteus vulgaris*	NO	YES
32	*Proteus mirabilis*	NO	YES
33	*Providencia alcalifaciens*	NO	YES
34	*Providencia rettgeri*	NO	NO
35	*Rahnella aquatilis*	YES	YES
36	*Raoultella planticola*	YES	YES
37	*Salmonella enterica subsp. enterica*	YES	YES
38	*Samsonia erythrinae*	NO	NO
39	*Serratia marcescens subsp. marcescens*	YES	YES
40	*Shigella dysenteriae*	YES	YES
41	*Shimwellia pseudoproteus*	NO	NO
42	*Sodalis glossinidius*	NO	NO
43	*Tatumella ptyseos*	NO	YES
44	*Trabulsiella guamensis*	YES	YES
45	*Thorsellia anophelis*	NO	NO
46	*Xenorhabdus bovieni*	NO	YES
47	*Yersinia enterocolitica subsp. enterocolitica*	YES	YES
48	*Yersinia pestis*	NO	YES
49	*Yokenella regensburgei*	YES	YES
50	*Wigglesworthia glossinidia*	NO	NO
	Summary	23 YES	37 YES

Table S4. Presence of an *ldcI* or *ldcC* gene in the 22 enterobacterial species found to contain *ldc*. Strains with a necessary correction of annotation are specified.

Supplementary Table S4

N°	Organism	*ldcI (cadA)*	*ldcC*	Strain with corrected annotation
1	*Buttiauxella agrestis*	NO	YES	
2	*Cedecea davisae*	NO	YES	
3	*Citrobacter freundii*	NO	YES	4_7_47CFA
4	*Cronobacter sakazakii*	NO	YES	NM1240
5	*Enterobacter cloacae subsp. cloacae*	NO	YES	ENHKU01
6	*Erwinia amylovora*	NO	YES	ATCC 49946
7	*Escherichia coli*	YES	YES	
8	*Ewingella americana*	NO	YES	
9	*Hafnia alvei*	YES	NO	FB1
10	*Klebsiella pneumoniae subsp. pneumoniae*	YES	YES	
11	*Kluyvera ascorbata*	YES	YES	ATCC 33433
12	*Leclercia adecarboxylata*	NO	YES	
13	*Pantoea agglomerans*	NO	YES	
14	*Plesiomonas shigelloides*	YES	YES	
15	*Rahnella aquatilis*	NO	YES	302-73
16	*Raoultella planticola*	NO	YES	ATCC 33071
17	*Salmonella enterica subsp. enterica*	YES	YES	OLF-SE9-10012
18	*Serratia marcescens subsp. marcescens*	YES	YES	ATCC 9150
19	*Shigella dysenteriae*	NO	YES	155-74
20	*Trabulsiella guamensis*	YES	YES	ATCC 49490
21	*Yersinia enterocolitica subsp. enterocolitica*	NO	YES	WA-314
22	*Yokenella regensburgei*	YES	YES	

Supplementary Movie S1. Superposition of the decameric structures colored as in the rest of the paper.
